# Modeling Spawning Habitats of *Coreius guichenoti* with Substrate Considerations: A Case Study of Pingdi Town in the Lower Jinsha River

**DOI:** 10.3390/ani15060881

**Published:** 2025-03-19

**Authors:** Wenchao Li, Dong Chen, Lekui Zhu, Tong Liu, Hanyue Wang, Litao Zhang, Rui Han, Zhi Yang, Jun Yan, Hongyi Yang, Anan Guo, Lei Liu

**Affiliations:** 1 College of Life Sciences, Shihezi University, Shihezi 832003, China; lwc1464347092@163.com (W.L.);; 2 Key Laboratory of Water Cycle and Related Land Surface Processes, Institute of Geographic Sciences and Natural Resources Research, Chinese Academy of Sciences, Beijing 100101, China; 3 College of Resources and Environment, University of Chinese Academy of Sciences, Beijing 100049, China; 4 School of Water Conservancy, North China University of Water Resources and Electric Power, Zhengzhou 450046, China; 5 State Key Laboratory of Simulation and Regulation of Water Cycle in River Basin, China Institute of Water Resources and Hydropower Research, Beijing 100038, China; 6 Institute of Hydroecology, Ministry of Water Resources and Chinese Academy of Sciences, Wuhan 430079, China; 7 Key Laboratory of Ecological Impacts of Hydraulic-Projects and Restoration of Aquatic Ecosystem of Ministry of Water Resource, Wuhan 430079, China

**Keywords:** lower Jinsha River, *Coreius guichenoti*, fuzzy logic, habitat, reproduction

## Abstract

*Coreius guichenoti*, once widely distributed in the upper reaches of the Jinsha River, has become a nationally protected species in China due to the profound impacts of cascade reservoirs. To assess the influence of substrate on the suitability of spawning habitat for *C. guichenoti*, this study develops a substrate-inclusive habitat model using fuzzy logic based on expert knowledge. Taking the Pingdi Town section of the lower Jinsha River—a historical spawning site for *C. guichenoti*—as a case study from March to July 2020, we simulated changes in the spawning habitat suitability index (HSI) and compared the results with those from traditional models that exclude substrate factors. The results showed that in the first and second halves of May, Weighted Usable Area (*WUA*) and Overall Suitability Index (OSI) increased by 42.31% and 38.73%, respectively, while MSP exhibited dramatic increases of 236.04% and 614.56%. These improvements were primarily observed along the riverbanks, where HSI increased by approximately 0.25. From a management perspective, the HSI results provide a scientific basis for optimizing ecological flow regulation. Incorporating substrate factors into spawning habitat models offers a more objective and comprehensive assessment of habitat quality. Habitat restoration measures, such as targeted substrate improvement in key riverbank areas, may further increase habitat suitability, providing additional opportunities for conservation planning in regulated rivers.

## Simple Summary

This study highlights the critical role of substrate in habitat modeling for *Coreius guichenoti*. Using a substrate-inclusive fuzzy logic model, we evaluated spawning habitat suitability in the Pingdi Town section of the lower Jinsha River from March to July 2020. The results showed that incorporating substrate factors significantly improved the habitat suitability assessment, particularly in riparian zones, better reflecting the actual spawning habitat preferences of *C. guichenoti* compared to traditional models. The spawning period spans from May to July, peaking in June, with suitable habitats mainly along riverbanks, which gradually contract over time. These findings provide key insights for conserving *C. guichenoti* populations and managing habitats affected by cascade reservoirs.

## 1. Introduction

Substrate is a crucial habitat factor for fish spawning, playing an essential role in their reproductive processes. Suitable substrate conditions significantly influence fish spawning behavior [1,2,3]. The microhabitats formed by riverbed substrates serve as hatching grounds for fish eggs, reducing the risk of injury during the hatching process. Furthermore, the larger gaps within the substrate provide refuge for fish, enhancing their survival [4,5,6,7]. The stability of the substrate also affects the growth of aquatic plants, which, in turn, influences fish feeding behavior [8].

Studies have shown that sediment accumulation and the burial of rock and gravel substrates following reservoir operation could be a key factor limiting the availability of spawning grounds [4]. For instance, high fine sediment accumulation rates negatively affect the hatching success of adhesive-spawning fish species, such as brook trout (*Salvelinus fontinalis*), rainbow trout (*Oncorhynchus mykiss*), Chinook salmon (*Oncorhynchus tshawytscha*), and nase (*Chondrostoma nasus*) [5,7].

As such, substrate is regarded as a key environmental factor in modeling fish spawning habitats and is commonly incorporated into habitat suitability index (HSI) models for fish habitat assessment [9,10]. The HSI framework provides a quantitative approach to evaluating habitat quality by integrating multiple environmental variables, including water velocity, depth, temperature, and substrate composition, to determine the suitability of a given habitat for fish reproduction [11,12].

In recent years, human activities have significantly altered the substrate structure of natural river habitats [13,14,15,16,17,18]. In the upper reaches of the Yangtze River, the operation of hydropower stations such as Xiangjiaba, Xiluodu, Baihetan, and Wudongde has profoundly impacted the substrate composition of riverbeds in reservoir sections [17,19,20,21,22,23]. Sediment accumulation in these areas has gradually transformed the substrate from natural coarse gravel to finer sediment layers, leading to a more uniform substrate distribution [20,22,23,24,25,26].

*Coreius guichenoti* is an endemic fish species in the upper Yangtze River, previously widely distributed in the Jinsha River basin. However, the construction and operation of cascade reservoirs have severely damaged its habitat, leading to a sharp decline in population size [27,28]. It has now been classified as a National Class II protected wild animal in China [29]. As a key indicator species for river ecosystem restoration, this fish holds significant research value in reservoir ecological regulation and habitat conservation [30,31]. Following the commissioning of the Wudongde Reservoir in 2020, the spawning habitats of *C. guichenoti* in the lower Jinsha River main stream are expected to face further degradation [28].

Field surveys indicate that the natural spawning grounds for *C. guichenoti* are predominantly located in river sections of the upper Yangtze River characterized by shoals, rapids, and gravel beds [27,32]. During the breeding season, the most suitable substrate types for spawning include small pebbles, large pebbles, and boulders [33]. However, the operation of cascade reservoirs causes sediment deposition in reservoir areas, resulting in smaller sediment particles covering or infiltrating the gravel layers [20,22,23,24,25,26]. This process significantly alters riverbed substrate types, thereby affecting the suitability of spawning habitats for *C. guichenoti*.

Despite the importance of substrate in fish habitat modeling, domestic studies on *C. guichenoti* habitat suitability have primarily focused on factors such as flow velocity and water depth, often neglecting the influence of substrate. For instance, Zhang et al. developed a spawning habitat model for *C. guichenoti* based on its preferences for water temperature, flow velocity, and water depth, and further predicted the impacts of climate change and hydropower operations on the species’ habitat [34]. Similarly, Wang et al. established a multi-objective habitat evaluation model for various fish species, including *C. guichenoti* and *Schizothorax prenanti*. This model, based on water depth and flow velocity preferences, identified an optimal ecological flow of 2395 m^3^/s for habitat protection within the “National Nature Reserve for Rare and Endemic Fish Species in the Upper Yangtze River” [35]. Liu et al. examined the effects of cascade barriers on the spawning and hatching processes of drifting egg species by investigating the flow velocity and water depth preferences of *C. guichenoti*, offering strategies for the protection of spawning habitats [36].

This study aims to address this research gap by developing a spawning habitat model for *C. guichenoti* that incorporates substrate requirements. The model simulates the spawning habitat conditions of *C. guichenoti* in the Pingdi Town section during the operational period of the Wudongde Reservoir from March to July 2020. The findings provide valuable recommendations for the conservation and management of *C. guichenoti* habitats.

## 2. Materials and Methods

### 2.1. Overview of the Study Site

**Figure 1:**
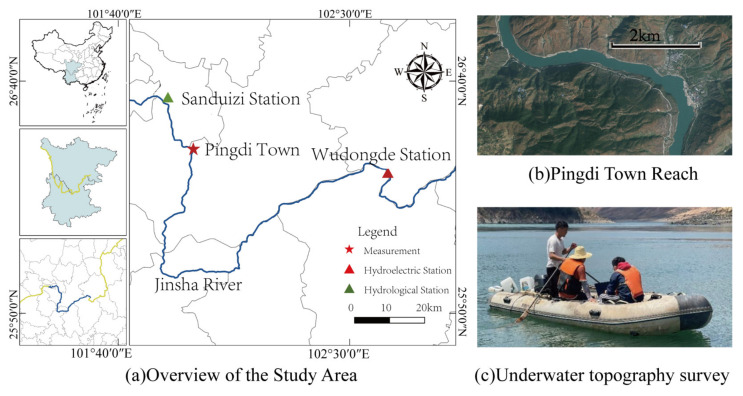
Schematic map of the study area. Note: In Figure (**a**), the blue line represents the target river segment and its adjacent reaches, while the red line indicates the actual course of the Jinsha River.

The study area is located in the Pingdi Town section of the main channel of the Jinsha River, covering a total length of 6 km. It is situated 18 km downstream of the Sanduizi hydrological station and 163 km upstream of the Wudongde Dam (Figure 1). This river section has historically served as a spawning ground for *C. guichenoti* [28]. When the Wudongde Reservoir reaches its normal storage level (975 m), the backflow will extend to the Sanduizi station [37], thereby affecting the Pingdi Town river section downstream of Sanduizi due to the reservoir’s backflow.

### 2.2. Habitat Model

Although the heterogeneity of expert knowledge may introduce bias into the model, this study adopts a fuzzy logic framework due to its unique ability to systematically integrate qualitative expertise. This approach effectively handles nonlinear and multivariable relationships while also addressing the challenges posed by data scarcity [12,38,39,40].

Based on existing data, this study builds upon the spawning habitat suitability model for *C. guichenoti* developed by Zhang et al. [34], which considers “water temperature”, “flow velocity”, and “water depth”, by incorporating substrate as an additional factor. The inclusion of substrate in this study is due to its more frequent association with *C. guichenoti* spawning grounds in field surveys compared to other influencing factors such as salinity and transparency. Moreover, numerous studies have demonstrated the species’ preference for specific substrates in its spawning habitat [27,32,33]. Therefore, adding the substrate factor has practical ecological significance rather than merely altering the model parameters.

The other three factors used in this study also have clear ecological significance and are widely applied in habitat models [12]. For instance, gonadal maturation requires a certain level of accumulated temperature [41], and after maturation, fish must reach a specific water temperature threshold to initiate spawning [33]. Additionally, spawning is often triggered by environmental stimuli such as flood pulses, water-level fluctuations, and changes in flow velocity [42,43].

**Figure 2:**
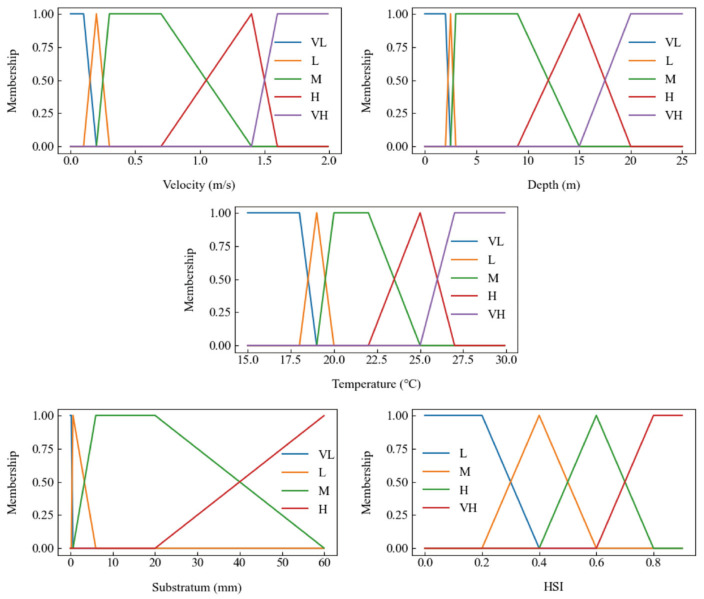
Fuzzy set and membership function of factors of the spawning habitat of *C. guichenoti*. Note: VL (Very Low), L (Low), M (Medium), H (High), VH (Very High).

The model incorporates four input factors: “water temperature”, “flow velocity”, “water depth”, and “substrate”. The output factor is the “habitat suitability index” (HSI). Each input factor is classified into fuzzy sets—“very low” (VL), “low” (L), “medium” (M), “high” (H), and “very high” (VH)—based on linguistic categories (Figure 2). The fuzzy sets often overlap with each other (Figure 2), indicating that a single value can simultaneously belong to multiple fuzzy sets. For example, when the water temperature is 17 °C, it falls entirely within the VL category. However, at 18 °C, there is a 50% probability of it belonging to VL and a 50% probability of it belonging to L.

The fuzzy set range is determined based on expert knowledge and existing ecological research. VL represents the lower temperature limit, VH represents the upper tolerance threshold, and M denotes the optimal spawning conditions. L and H represent moderately suitable spawning conditions. For example, regarding water temperature, based on field measurements and expert experience [33,44], water temperatures below 18 °C or above 27 °C are considered unsuitable for spawning, while temperatures between 20 and 25 °C are optimal. Temperatures ranging from 18 to 20 °C and from 25 to 27 °C are considered moderately suitable.

The fuzzy rules consist of two parts: one describes the habitat conditions, and the other corresponds to the HSI. For example, under conditions where the substrate is medium, water temperature is high, flow velocity is high, and water depth is medium, the HSI is considered medium (Table 1).

Based on the work of Zhang Peng et al. [34], substrate was added to establish a fuzzy rule base. The established fuzzy rules take into account the following:

1. Threshold Requirements for Habitat Factors: The spawning of *C. guichenoti* requires specific environmental conditions. For example, spawning activity is minimal or does not occur when the water temperature is below 18 °C.
2. Complementary and Trade-off Effects of Habitat Factors: Once all habitat factors meet the threshold requirements, their influence on spawning suitability may exhibit complementary or trade-off relationships. For instance, even if water temperature suitability is relatively low, the habitat may still be considered suitable for *C. guichenoti* spawning if flow velocity, water depth, and substrate conditions are highly favorable.
3. Relative Importance of Habitat Factors: When assessing complementary or trade-off effects, different habitat factors have varying degrees of importance, ranked as follows: water temperature > flow velocity > water depth > substrate grain size.

The fuzzy rules are defuzzified using the centroid method, and the specific calculation method is as follows:

(1) $$HSI=\int_{Z_{min}}^{Z_{max}}Z\mu_{c}\left(Z\right)d_{Z}/\int_{Z_{min}}^{Z_{max}}\mu_{c}\left(Z\right)d_{Z}$$

In the formula, *Z* is the crisp value of the fuzzy set, *Z_min_* and *Z_max_* are the minimum and maximum values of *Z*, *μ_c_*(*z*) is the membership function of fuzzy set *C*, and HSI is the final crisp value obtained after defuzzification.

### 2.3. Habitat Simulation

The bed surface particle size can be estimated by reverse-calculating the substrate type using the threshold velocity equation [45]:

(2) $$U_{e}=({\frac{h}{d})}^{0.14}{\left[17.6\frac{\rho_{s}-\rho }{\rho }d+\frac{0.000000605\left(10+h\right)}{d^{0.72}}\right]}^{0.5}$$

In the formula, *U_e_* is the critical velocity (m/s), *h* is the water depth (m), *d* is the sediment diameter (m), *ρ_s_* is the sediment density (2.65 g/cm^3^), and *ρ* is the water density (1 g/cm^3^).

**Figure 3:**
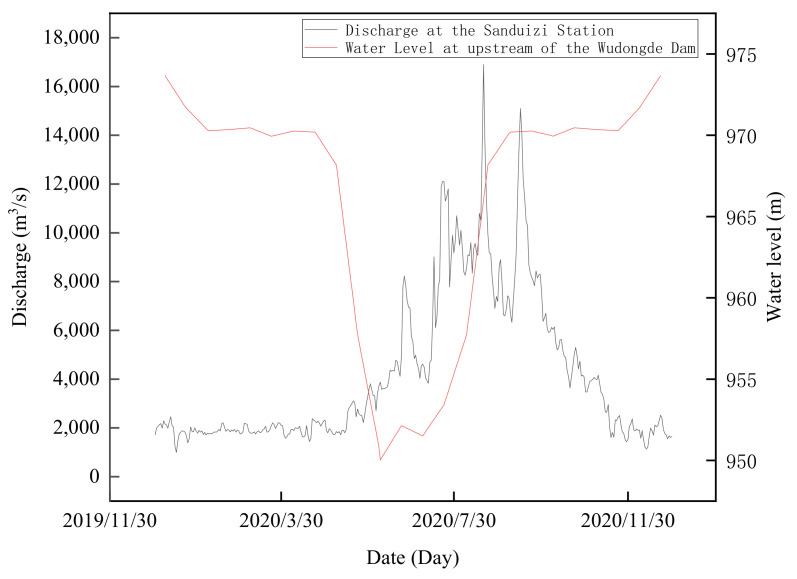
Discharge at Sanduizi Station and water level in front of Wudongde Dam in 2020 [46,47].

A one-dimensional and two-dimensional coupled hydrodynamic model with a semi-monthly time step was developed for this study. Boundary data were obtained from the flow measurements at the Sanduizi hydrological station and the observed water level at the Wudongde Dam (Figure 3). The model conditions and boundaries are summarized in Table 2.

The one-dimensional hydrodynamic model was developed for the 197 km stretch between San Duizi and Wudongde, using the Preissmann four-point implicit scheme to discretize the governing equations. This model provided the upstream and downstream boundary conditions for the two-dimensional hydrodynamic model applied to the Pingdi Town river section.

**Figure 4:**
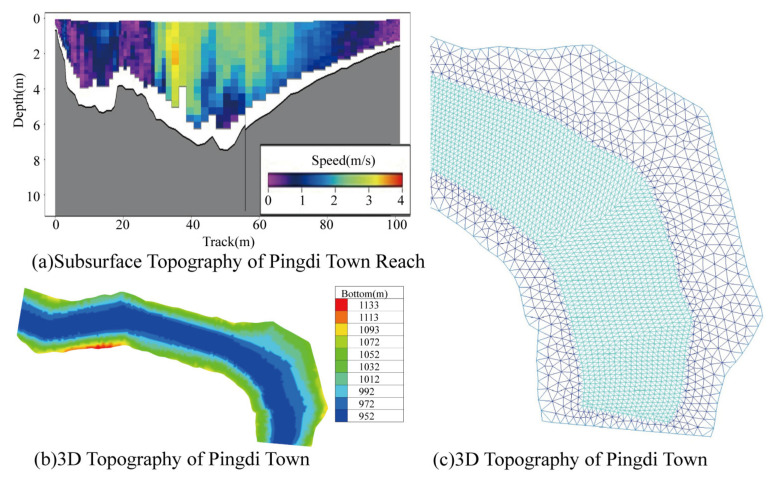
Schematic diagram of two-dimensional hydrodynamic model principles. Note: In Figure (**a**), the color gradient from blue to red represents the flow velocity increasing from 0 m/s to 4 m/s. In Figure (**b**), the color gradient from blue to red indicates the elevation rising from 952 m to 1133 m. In Figure (**c**), the color variation is associated with grid density, with darker colors representing higher density.

The two-dimensional hydrodynamic model for the Pingdi Town river section was established using the TELEMAC-MASCARET software v8.3 (EDF R&D, Chatou, Île-de-France, France), incorporating the measured underwater topography (Figure 4a) and riverbank topography (Figure 4b) of the study area. An unstructured triangular mesh was employed, with a grid spacing of 20 m in the river channel and 50 m on the floodplain, resulting in a total of 172,074 grids (Figure 4c). The model was validated using actual water level measurements from the Longjie water level station [46].

### 2.4. Evaluation of Spawning Habitat for C. guichenoti

For the spawning grounds of *C. guichenoti*, a larger suitable spawning area indicates higher habitat suitability, providing more opportunities for the target fish to spawn. The habitat condition or availability of the entire river section is quantitatively evaluated using several indices, including the weighted usable area (WUA), overall suitability index (OSI), ideal habitat proportion (ISP), moderate habitat proportion (MSP), and low-suitability habitat proportion (LSP). The calculation methods are as follows [49]:

(3) $$WUA=\sum_{i=1}^{N}A_{i}{HSI}_{i}$$

(4) $$OSI=\frac{\sum_{i=1}^{N}A_{i}{HSI}_{i}}{\sum_{i=1}^{N}A_{i}}$$

(5) $$ISP=\frac{\sum_{i=1}^{N}A_{i}{(HSI}_{i}\geq 0.7)}{\sum_{i=1}^{N}A_{i}}\times 100\%$$

(6) $$MSP=\frac{\sum_{i=1}^{N}A_{i}{(0.3\leq HSI}_{i}<0.7)}{\sum_{i=1}^{N}A_{i}}\times 100\%$$

(7) $$LSP=\frac{\sum_{i=1}^{N}A_{i}{(HSI}_{i}<0.3)}{\sum_{i=1}^{N}A_{i}}\times 100\%$$

In the formula, WUA is the weighted usable area (m^2^), *A_i_* is the area of the *i*-th cell in the two-dimensional hydrodynamic model grid (m^2^), HSI_i_ is the habitat suitability index in the *i*-th cell, and *N* is the total number of grid cells in the model.

### 2.5. Influence of Substrate on Model Performance

A comparison was made between the spawning habitat model for *C. guichenoti* established by Zhang Peng et al. [44], which does not account for substrate type, and the model developed in this study, which incorporates substrate factors. Both models were applied to the same river section and time period. The differences between the models that consider substrate type and those that do not were analyzed.

## 3. Results

### 3.1. Validation and Results of the Hydrodynamic Model

**Figure 5:**
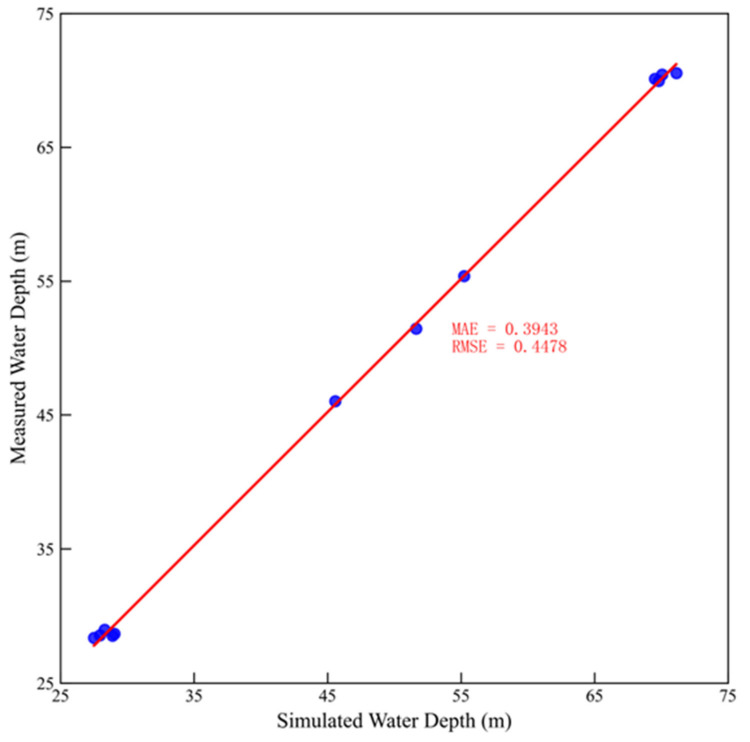
Comparison of simulated and measured water depth at Longjie Station. Note: The red line represents the measured water depth, the blue dots indicate the simulated water depth.

The one-dimensional hydrodynamic model was validated using observed water levels from the Longjie Station, recorded on the first day of each month from March to July [34], with Manning’s coefficients listed in Table 1. Figure 5 presents a comparison between the simulated and observed water depths. The comparison reveals a good agreement between the simulation parameters and the observed results. The mean absolute error (MAE) for the simulated water levels was 0.3943 m, and the root mean square error (RMSE) was 0.4478 m.

**Figure 6:**
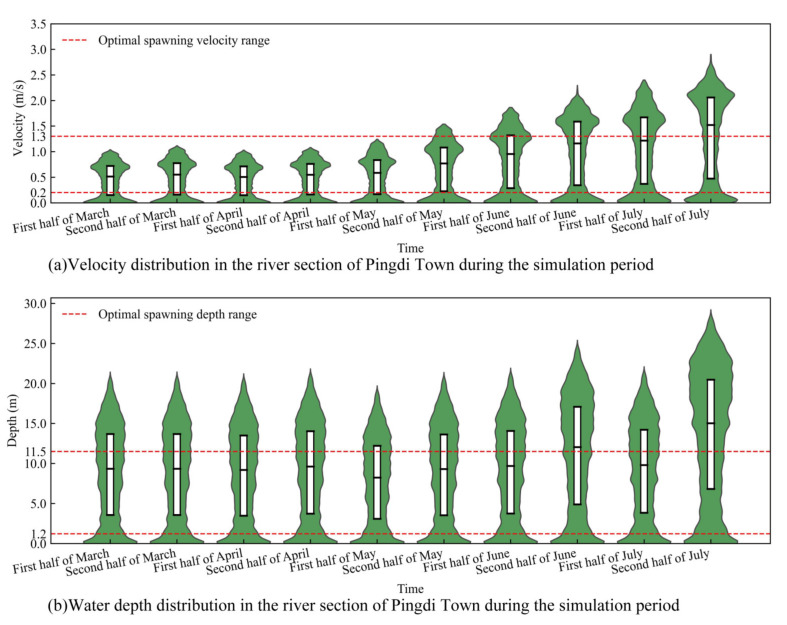
Distribution of velocity in the river section of Pingdi Town over simulated time.

The results from the hydrodynamic model (Figure 6) show that from early March to mid-May 2020, due to relatively low upstream discharge (1840.67–2066.67 m^3^/s), the average water depth in the Pingdi Town river section ranged from 9 to 10 m, which is within the optimal spawning depth range for *C. guichenoti* (1.2–11.5 m) [33]. The average flow velocity in the river channel was between 0.4 and 0.6 m/s, also within the optimal spawning flow velocity range (0.2–1.3 m/s) [33,50]. However, from late May to late July, as the flood season began, upstream discharge increased rapidly (by 242.11%), peaking at 9596.25 m^3^/s in late July. During this period, approximately 60% of the river section had a depth greater than 15 m, and more than 50% of the section had a flow velocity exceeding 1.5 m/s, conditions that surpassed the optimal spawning parameters for *C. guichenoti*.

**Figure 7:**
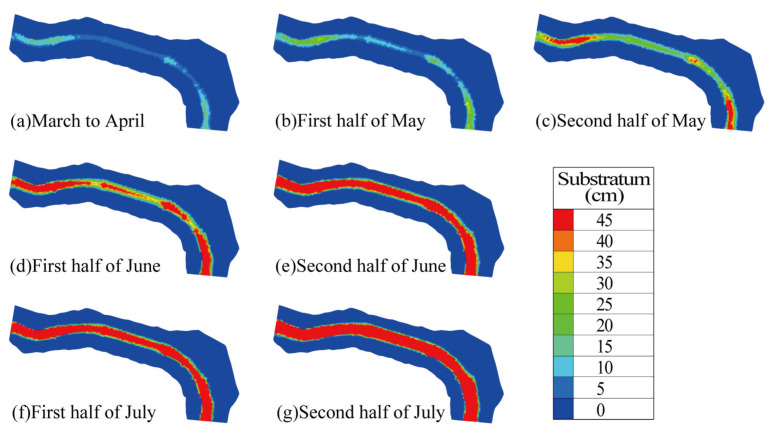
Distribution of bed sediment grain size in the river section of Pingdi Town over simulated time.

As shown in Figure 7, the simulated substrate grain size in the Pingdi Town river section exhibited a general increasing trend over time. From early March to mid-May, 80% of the river section had substrate grain sizes smaller than 25 cm, consisting mainly of gravel and cobble, which are suitable for *C. guichenoti* spawning [33,50]. However, from June to July, the majority of the river section had substrate grain sizes exceeding 40 cm, classified as boulders, which resulted in a decrease in spawning suitability for *C. guichenoti*.

### 3.2. Evaluation of Spawning Habitat for C. guichenoti After Reservoir Impoundment at the Wudongde Hydropower Station

**Figure 8:**
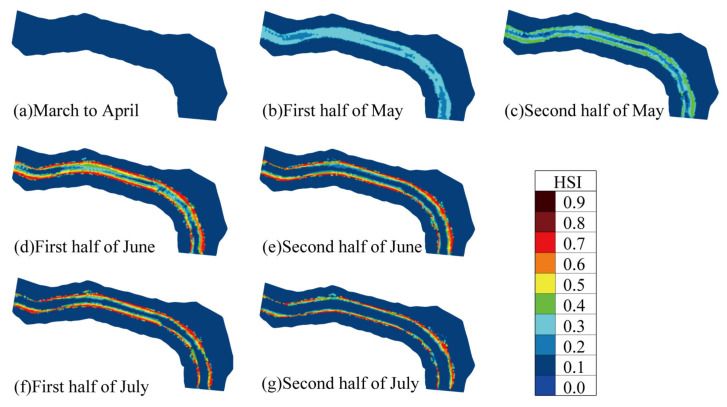
Distribution of HSI in the river section of Pingdi Town over simulated time.

The simulation results indicate that from March to April, the entire reach was unsuitable for spawning, with the habitat suitability index (HSI) for the reach falling below 0.2. During this period, the low-suitability habitat proportion (LSP) was 1, while the weighted usable area (WUA) and overall suitability index (OSI) were approximately 2.4 × 10^5^ m^2^ and 0.06, respectively. Between May and July, the area of suitable spawning habitats increased, peaking in early June. At that time, the WUA and OSI reached 6.8 × 10^5^ m^2^ and 0.1547, respectively. The ideal habitat proportion (ISP), moderate habitat proportion (MSP), and low-suitability habitat proportion (LSP) were 3.81%, 20.68%, and 75.51%, respectively, indicating that the reach provided the most suitable spawning area between May and July, with June having the largest suitable area (Table 3, Figure 8). This finding aligns with the simulation results of Zhang et al. [44,51] for spawning grounds near Panzhihua upstream of Pingdi Town on the Jinsha River, where the time of the largest suitable spawning habitat area coincides with early field resource survey results [28].

The increase in suitable spawning habitat area from May to July mainly resulted from the rise in medium-suitability area proportions, followed by an increase in ideal habitat area proportions. Compared to 0% in March and April, the proportion of medium-suitability habitats exceeded 13% from May to July, peaking at 22.58% in early May. The proportion of ideal habitats was 0% from March to May, peaked at 3.81% in early June, and then decreased over time (Table 3).

The simulation results show that suitable spawning areas for *C. guichenoti* were primarily concentrated in the riparian zones. After early June, these areas gradually shrank towards the riverbanks (Figure 8). From May to July, the HSI of riparian zones was higher than that of the river center, particularly in June and July, when medium and ideal habitats were primarily distributed along the banks. Over time, suitable spawning areas shrank, but the HSI of these areas increased. In early May, the average HSI of suitable spawning areas was 0.3, increasing to 0.4 in late May. In June and July, the HSI ranged from 0.4 to 0.8 (Figure 8). This pattern can be attributed to water temperature. In May, the water temperature of 18.7 °C was below the optimal spawning temperature range for *C. guichenoti* (20–25.2 °C) [33,50], resulting in low suitability for spawning. In June and July, water temperatures were 20.6 °C and 21.1 °C, respectively, falling within the optimal range for spawning. However, the flood process caused excessive flow velocity in the river center, making it unsuitable for spawning.

### 3.3. The Impact of InIncorporating Substrate into the Model

**Figure 9:**
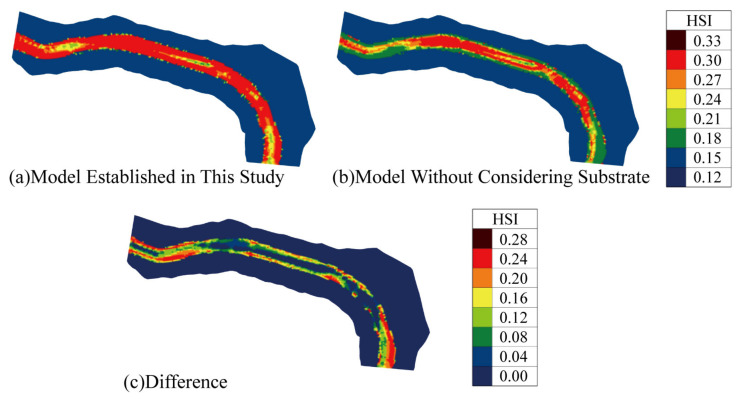
Comparison of HSI for two models during the period of maximum simulation difference (early May).

Table 4 highlights the differences in simulated habitat quality for the Pingdi Town reach between the model developed in this study and a model that does not consider substrate [44]. Significant differences were observed in May, with the model developed in this study showing a substantial increase in suitable spawning areas for *C. guichenoti* in the Pingdi Town reach. This increase was primarily attributed to an expansion in medium-suitability habitat areas and a reduction in unsuitable habitat areas, particularly in riparian zones (Table 4, Figure 9).

In early May, the weighted usable area (WUA) and overall suitability index (OSI) increased by 42.31%, the moderate suitability habitat proportion (MSP) increased by 236.04%, and the low-suitability habitat proportion (LSP) decreased by 17.00%. In late May, the WUA and OSI increased by 38.73%, the MSP increased by 614.56%, and the LSP decreased by 16.3% (Table 4).

## 4. Discussion

### 4.1. The Necessity and Ecological Significance of Including Substrate in the Model

Due to the lack of field data, this study did not validate the habitat model. However, the model still provides valuable insights into the unique substrate preferences of *C. guichenoti* and underscores the necessity of incorporating substrate factors into habitat models. Model bias arises from the heterogeneity of expert knowledge. To minimize model errors, this study adopts a fuzzy logic framework, which integrates extensive expert experience. The model’s unique ability to systematically incorporate qualitative expert knowledge enables it to effectively address nonlinear and multivariable relationships, as well as challenges posed by data scarcity [12,38,39,40].

Fish exhibit specific substrate preferences for their spawning habitats. For example, *C. guichenoti* prefers spawning substrates such as gravel and boulders [33], whereas the “four major carp” species tend to favor sandy or mud–sand mixed substrates [52]. Suitable substrate conditions facilitate egg attachment and hatching, provide shelter, and influence feeding [4,5,6,7,8]. Thus, substrate is considered a crucial factor in fish spawning [9,10]. However, many traditional habitat models primarily focus on variables like flow velocity, water depth, and temperature, often neglecting the role of substrate, which could lead to biased habitat suitability assessments [34,52].

The results of this study demonstrate that in the Pingdi Town river section, habitat quality in May significantly improved after accounting for substrate (the WUA increased by 42.31% and 38.73% in the first and second halves of May, respectively). This suggests not only that substrate is an important factor affecting habitat suitability but also that it may play a critical role in enhancing habitat quality under temperature constraints. Similar studies have shown that substrate alone can account for up to 27.8% of the habitat preferences of species such as *Schizothorax prenanti* [9].

Furthermore, the simulation results indicate that the substrates suitable for *C. guichenoti* spawning are primarily cobbles and boulders, with corresponding flow velocities ranging from 0.75 to 2 m/s and water depths exceeding 5 m. These characteristics align closely with the natural spawning sites, which are typically gravel bars and riffles dominated by cobbles and boulders, characterized by deep water, swift flows, and complex flow patterns [27,32,33,53].

Therefore, incorporating substrate into the model not only aligns with international trends in ecological research but also refines the quantitative expression of habitat suitability in this study.

### 4.2. The Importance and Impact of Other Environmental Factors in the Model

The other three factors used in this study also play a crucial ecological role in the spawning process of *C. guichenoti*.

Water temperature is a key environmental factor determining the initiation of fish spawning. The critical spawning temperature for *C. guichenoti* is approximately 18 °C, with an optimal range of 20–22 °C [44]. From March to April, the water temperature at the Wudongde Reservoir remained below 18 °C, preventing spawning. By May, the temperature increased to 18.7 °C, leading to an overall increase in the habitat suitability index (HSI) in the study reach, but habitat quality remained relatively low. In June and July, when the temperature reached the optimal spawning range, the HSI values were highest along the riverbanks.

The optimal spawning depth for *C. guichenoti* ranges from 1.2 to 11.5 m, while the preferred flow velocity is 0.2–1.3 m/s [33]. Although water depth and flow velocity were suitable in May, the relatively low water temperature resulted in lower WUA and MSP values. From May to July, as discharge increased and water levels dropped, flow velocity in the river center increased significantly, surpassing the optimal spawning velocity range for *C. guichenoti* (0.2–1.3 m/s) [33]. Consequently, the suitable spawning areas shifted from the river center towards the riverbanks over time.

Additionally, *C. guichenoti* produces drifting eggs that must remain suspended within a specific flow velocity range to ensure successful development and prevent settlement, which could lead to embryonic asphyxiation [18]. Research indicates that the critical velocity range for safe egg drift is 0.2–0.7 m/s [54,55,56,57]. If the velocity drops below this threshold, eggs are likely to settle on the riverbed, where they risk being buried by sediment or suffering from oxygen depletion, ultimately reducing hatching success [58,59]. On the other hand, excessive turbulence may disperse the eggs into unsuitable habitats or increase the likelihood of mechanical damage, further compromising their survival.

### 4.3. Other Factors Affecting Spawning and Model Application

**Figure 10:**
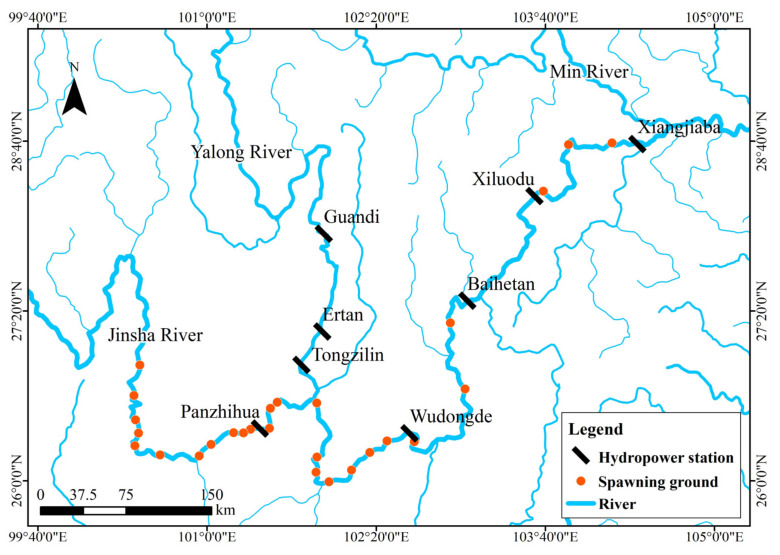
Spawning grounds of *C. guichenoti* [28].

Figure 10 shows the historical distribution of spawning grounds for *C. guichenoti* in the main stream from 2012 to 2021.

*C. guichenoti* is a typical migratory species that is influenced by a variety of environmental factors, particularly the connectivity of water flow. The connectivity of water flow is crucial for fish migration, as it not only affects the movement of fish between water bodies but also plays a key role in their ability to access suitable spawning grounds. The construction of cascaded reservoirs has altered the natural flow patterns of water, severing fish migration routes and preventing fish from reaching resource-rich upstream areas, thus impacting their breeding and early resource replenishment [28].

Seasonal changes also have a complex effect on the suitability of fish habitats [34]. In this study, due to the influence of water temperature, the habitat suitability for *C. guichenoti* peaked in June. Some studies have shown that after the cascade reservoirs store water, the spawning time of *C. guichenoti* may be delayed. Moreover, seasonal fluctuations in factors such as water temperature, flow speed, and water depth have a profound impact on habitat suitability. Therefore, relying solely on short-term observations is insufficient, and a longer time scale is needed to analyze how these factors influence spawning success rates, particularly in terms of fish migration timing and spawning periods. The operational mode of reservoirs, especially the way water discharge is regulated, may lead to significant changes in hydrological conditions, which directly affect fish migration and spawning times. Hydrological changes and the operating mode of reservoirs should be a key focus of future research [28,34].

The findings of this study provide theoretical support for ecological flow management and habitat protection strategies. Specifically, the study indicates that June and July are the best spawning months for *C. guichenoti*, but if the upstream reservoir discharges too much water and the flow velocity is too high, it can cause the flow speed in the river center to become excessive, thus reducing the suitability of the spawning grounds. Therefore, to address this issue, reservoir operations can be appropriately adjusted, such as by reducing upstream discharge or storing water in advance to regulate the flow velocity and restore the suitability of fish habitats. Additionally, habitat restoration measures, such as improving the substrate in key river sections, can further enhance habitat suitability. These measures will not only improve the spawning success rate of *C. guichenoti* but also provide effective references for ecological protection planning in regulated rivers.

## 5. Conclusions

This study establishes a spawning habitat model for *C. guichenoti* that incorporates substrate considerations, based on field survey data and expert knowledge. Using the Pingdi Town section of the lower Jinsha River from March to July 2020 as a case study, the impact of substrate on the spawning habitat model was analyzed. The results show that:

1. Compensatory Effect of Substrate: Substrate plays a compensatory role in the spawning of *C. guichenoti*. Compared to traditional models, the model developed in this study provided a higher habitat quality evaluation for May. Specifically, the WUA and OSI increased by 42.31% and 38.73% in the first and second halves of May, respectively, while the MSP increased by 236.04% and 614.56%. These increases were mainly observed in the riverbank areas, with the HSI rising by approximately 0.25.
2. Necessity of Substrate Inclusion: It is essential to incorporate substrate into the spawning habitat model for *C. guichenoti*. The model developed in this study effectively reflects the complex habitat requirements for the species’ spawning. It is a valuable tool for quantifying and assessing the spawning habitat of *C. guichenoti* in the study area.
3. Impact of Wudongde Reservoir Impoundment: Following the impoundment operations of the Wudongde Reservoir, *C. guichenoti* spawning in the downstream Pingdi Town reach of the Jinsha River occurred from May to July, with June being the peak spawning period. Suitable spawning areas were primarily concentrated along the riverbanks and progressively shrank towards the riverbanks as time went on.

Due to the lack of field-measured spawning data after the impoundment operation of the Wudongde Reservoir, this study is currently unable to validate the habitat model considering substrate conditions. Therefore, future research should conduct systematic field baseline data surveys to support further model optimization and validation.

## Figures and Tables

**Table 1:** Fuzzy rules of spawning habitat for *C. guichenoti*.

Substrate	Temperature	Velocity	Depth	HSI
L/M/H	M	L/H	L/H	M
L/M/H	L/H	M	L/H	M
M	L/H	L/H	M	M
L/H	L/H	L/H	M	L
L/M/H	M	M	L/H	H
L/M/H	M	L/H	M	H
M	L/H	M	M	H
L/H	L/H	M	M	M
L/M/H	M	M	M	VH
VL	AC	AC	AC	L
L/M/H	VL/VH	AC	AC	L
L/M/H	L/M/H	VL/VH	AC	L
L/M/H	L/M/H	L/M/H	VL/VH	L

Note: VL (very low), L (low), M (medium), H (high), VH (very high). AC represents the entire fuzzy set of the factor. The “/” symbol indicates “or.” Each row represents a combination of four environmental factors—substrate, temperature, velocity, and depth—and their corresponding habitat suitability index (HSI) range. For example, when substrate is L, M, or H; temperature is M; velocity is L or H; and depth is L or H, the resulting HSI is M.

**Table 2:** Setup of hydrodynamic model operating conditions.

Time	Discharge (m^3^/s)	Water Level (m)	Manning Coefficient	Temperature (°C)
First half of March	1884	970.46	0.1780	13.40
Second half of March	2023.63	969.94	0.1780	13.40
First half of April	1824	970.26	0.1810	16.10
Second half of April	2066.67	970.19	0.1810	16.10
First half of May	1840.67	968.17	0.1704	18.70
Second half of May	2805	957.74	0.1704	18.70
First half of June	3610.67	950.03	0.1071	20.60
Second half of June	5778.67	952.14	0.1071	20.60
First half of July	4654.67	951.51	0.0883	21.10
Second half of July	9596.25	973.66	0.0883	21.10

Note: The water temperature data come from the actual measured water temperature at the tail of the Wudongde reservoir [48].

**Table 3:** March–July spawning habitat quality assessment of *C. guichenoti*.

Time	WUA (10^5^ m^2^)	OSI	ISP	MSP	LSP
March to April	2.41	0.06	0.00%	0.00%	100.00%
First half of May	4.80	0.11	0.00%	22.58%	77.42%
Second half of May	4.70	0.11	0.00%	18.47%	81.53%
First half of June	6.79	0.15	3.81%	20.68%	75.51%
Second half of June	6.11	0.14	2.72%	16.37%	80.92%
First half of July	5.59	0.13	2.63%	14.89%	82.48%
Second half of July	5.40	0.12	1.29%	13.07%	85.63%

**Table 4:** The difference in simulation results between the two models.

Time	WUA (10^5^ m^2^)	OSI	ISP	MSP	LSP
March to April	0.00%	0.00%	0.00%	0.00%	0.00%
First half of May	42.31%	42.31%	0.00%	236.04%	−17.00%
Second half of May	38.73%	38.73%	0.00%	614.56%	−16.30%
First half of June	−0.13%	−0.13%	−1.68%	0.21%	0.03%
Second half of June	−0.17%	−0.17%	−1.74%	−0.18%	0.10%
First half of July	−0.16%	−0.16%	−1.33%	−0.26%	0.09%
Second half of July	−0.19%	−0.19%	−3.01%	−0.46%	0.12%

Note: The values in the table indicate the relative increase (%) after incorporating substrate into the model.

## Data Availability

Data are contained within the article.

## References

[1] McAdam D.S.O. (2015). Retrospective weight-of-evidence analysis identifies substrate change as the apparent cause of recruitment failure in the upper Columbia River white sturgeon (*Acipenser transmontanus*). Can. J. Fish. Aquat. Sci..

[2] Baril A.-M., Buszkiewicz J.T., Biron P.M., Phelps Q.E., Grant J.W. (2018). Lake sturgeon (*Acipenser fulvescens*) spawning habitat: A quantitative review. Can. J. Fish. Aquat. Sci..

[3] Wang P., Shen Y., Wang C., Hou J., Qian J., Yu Y., Kong N. (2017). An improved habitat model to evaluate the impact of water conservancy projects on Chinese sturgeon (*Acipenser sinensis*) spawning sites in the Yangtze River, China. Ecol. Eng..

[4] Du H., Wei Q., Zhang H., Liu Z., Wang C., Li Y. (2011). Bottom substrate attributes relative to bedform morphology of spawning site of Chinese sturgeon *Acipenser sinensis* below the Gezhouba dam. J. Appl. Ichthyol..

[5] Duerregger A., Pander J., Palt M., Mueller M., Nagel C., Geist J. (2018). The importance of stream interstitial conditions for the early-life-stage development of the European nase (*Chondrostoma nasus* L.). Ecol. Freshw. Fish.

[6] Merz J.E., Setka J.D., Pasternack G.B., Wheaton J.M. (2004). Predicting benefits of spawning-habitat rehabilitation to salmonid (*Oncorhynchus* spp.) fry production in a regulated California river. Can. J. Fish. Aquat. Sci..

[7] Chapman J.M., Proulx C.L., Veilleux M.A., Levert C., Bliss S., Andre M.-E., Lapointe N.W., Cooke S.J. (2014). Clear as mud: A meta-analysis on the effects of sedimentation on freshwater fish and the effectiveness of sediment-control measures. Water Res..

[8] Hou Y., Yang Z., An R., Cai L., Chen X., Zhao X., Zou X. (2019). Water flow and substrate preferences of Schizothorax wangchiachii (Fang, 1936). Ecol. Eng..

[9] Yao W. (2021). Ecohydraulic tools for aquatic fauna habitat and population status assessment, analysis and monitoring aimed at promoting integrated river management. Ecol. Model..

[10] Wang Q., Han Y., Li P., Zhang W., Wang Y., Xi Y., Yao W. (2023). Ecohydraulic modelling to evaluate cascade dam construction impact and support fish habitat restoration. Ecol. Eng..

[11] Armour C.L., Taylor J.G. (1991). Evaluation of the instream flow incremental methodology by US Fish and Wildlife Service field users. Fisheries.

[12] Yi Y., Zhang S. (2019). Review of aquatic species habitat simulation method and modelling. Sci. Sin. Technol..

[13] Chen Q., Li Q., Lin Y., Zhang J., Xia J., Ni J., Cooke S.J., Best J., He S., Feng T. (2023). River Damming Impacts on Fish Habitat and Associated Conservation Measures. Rev. Geophys..

[14] He F., Zarfl C., Tockner K., Olden J.D., Campos Z., Muniz F., Svenning J.-C., Jähnig S.C. (2024). Hydropower impacts on riverine biodiversity. Nat. Rev. Earth Environ..

[15] Csiki S.J., Rhoads B.L. (2014). Influence of four run-of-river dams on channel morphology and sediment characteristics in Illinois, USA. Geomorphology.

[16] Kuriqi A., Pinheiro A.N., Sordo-Ward A., Bejarano M.D., Garrote L. (2021). Ecological impacts of run-of-river hydropower plants—Current status and future prospects on the brink of energy transition. Renew. Sustain. Energy Rev..

[17] Li T., Wang S., Liu Y., Fu B., Zhao W. (2018). Driving forces and their contribution to the recent decrease in sediment flux to ocean of major rivers in China. Sci. Total Environ..

[18] Wenxuan C., Jianbo C., Ye Q., Zhonghua D. (2007). Fish Resources of Early Life History Stagrs in Yangze River.

[19] Li D., Lu X.X., Yang X., Chen L., Lin L. (2018). Sediment load responses to climate variation and cascade reservoirs in the Yangtze River: A case study of the Jinsha River. Geomorphology.

[20] Zhu L., Chen C., Zhang J. (2016). Study on variations of runoff and sediment and effect to the lower Jinsha River. J. Sediment Res..

[21] Zhu L., Chen D., Yang C., Chen K., Li S. (2023). Sediment dep*OSI*tion of cascade reservoirs in the lower Jinsha River and scouring of river channel under dam. J. Lake Sci..

[22] Du Z., Dong X., Zhang F., Qin L. (2022). Study on runoff and sediment characteristics and reservoir dep*OSI*tion in Xiluodu Reservoir of the Jinsha River. J. Sediment Res..

[23] Yue L. (2021). The Evolution of Flow and Sediment Regime in the Yangtze River and Its Fish Response Mechanism. Master’s Thesis.

[24] Liu S., Li D., Liu D., Zhang X., Wang Z. (2022). Characteristics of sedimentation and sediment trapping efficiency in the Three Gorges Reservoir, China. Catena.

[25] Rodriguez A., McKee B., Miller C., Bost M., Atencio A. (2020). Coastal sedimentation across North America doubled in the 20th century despite river dams. Nat. Commun..

[26] Csiki S., Rhoads B.L. (2010). Hydraulic and geomorphological effects of run-of-river dams. Prog. Phys. Geogr..

[27] Lehe L., Guoxi W., Zhiling W. (1990). Reproduction ecology of *Coreius heterodon* (Bleeker) and *Coreius guichenoti* (Sauvage et Dabry) in the mainstream of the Changjiang River after the construction of *Gezhouba Dam*. Acta Hydrobiol. Sin..

[28] Meihua X., Ke S., Weitao L., Bin Z. (2023). Research progress on resources variation and protection of *Coreius guichenoti*. Yangtze River.

[29] Jiang Z., Jiang J., Wang Y., Zhang E., Zhang Y., Li L., Xie F., Cai B., Cao L., Zheng G. (2016). Red List of China’s Vertebrates. Biodivers. Sci..

[30] Ren Y., Zhao L., Cao H., Ruan Y. (2020). Influence of ecological regulation of cascade reservoirs in the lower Jinsha River. Ecol. Environ. Monit. Three Gorges.

[31] Zhang D., Fan H., Wang M., Li F., Ruan Y. (2022). Target Fish Screening for the Ecological Operation of Wudongde Hydropower Station on Jinsha River. J. Hydroecology.

[32] Yichao Z. (2009). Impactof Dam on Natural Reproduction of *Coreius guichenoti* and *Rhinogobio ventralis* in Upstream of Yangtze River. Ph.D. Thesis.

[33] Yang Z., Zhang P., Tang H., Gong Y., Dong C., Chen X., Zhao N. (2017). The formation of habitat suitability curves for *Coreius guichenoti* (Sauvage & Dabry de Thiersant,1874) of the lower Jinsha River. Ecol. Sci..

[34] Zhang P., Qiao Y., Schineider M., Chang J., Mutzner R., Fluixa-Sanmartin J., Yang Z., Fu R., Chen X., Cai L. (2019). Using a hierarchical model framework to assess climate change and hydropower operation impacts on the habitat of an imperiled fish in the Jinsha River, China. Sci. Total Environ..

[35] Wang H., Wang H., Hao Z., Wang X., Liu M., Wang Y. (2018). Multi-objective assessment of the ecological flow requirement in the upper Yangtze national nature reserve in China using PHABSIM. Water.

[36] Liu Q., Zhang P., Li H., You L., Li Y., Li J., Liu M., Zhao P., Wang K., Zhu Z. (2021). Assessment and conservation strategies for endemic fish with drifting eggs threatened by the cascade barrier effect: A case study in the Yalong River, China. Ecol. Eng..

[37] Ruihua S., Anxiu P. (2023). Variation of Water Level-discharge Relationship in Sanduizi Hydrological Station after Impoundment of Wudongde Power Station. Ecol. Environ. Monit. Three Gorges.

[38] Broekhoven E.V., Adriaenssens V., Baets B.D., Verdonschot P.F.M. (2006). Fuzzy rule-based macroinvertebrate habitat suitability models for running waters. Ecol. Model..

[39] Jorde K., Schneider M., Peter A., Zoellner F. Fuzzy based models for the evaluation of fish habitat quality and instream flow assessment. Proceedings of the 3rd International Symposium on Environmental Hydraulics.

[40] Theodoropoulos C., Vourka A., Skoulikidis N., Rutschmann P., Stamou A. (2018). Evaluating the performance of habitat models for predicting the environmental flow requirements of benthic macroinvertebrates. J. Ecohydraulics.

[41] Li T., Mo K., Wang J., Chen Q., Zhang J., Zeng C., Zhang H., Yang P. (2021). Mismatch between critical and accumulated temperature following river damming impacts fish spawning. Sci. Total Environ..

[42] Young P.S., Cech J.J., Thompson L.C. (2011). Hydropower-related pulsed-flow impacts on stream fishes: A brief review, conceptual model, knowledge gaps, and research needs. Rev. Fish Biol. Fish..

[43] Chen Q., Zhang J., Chen Y., Mo K., Wang J., Tang L., Lin Y., Chen L., Gao Y., Jiang W. (2021). Inducing flow velocities to manage fish reproduction in regulated rivers. Engineering.

[44] Zhang P., Yang Z., Cai L., Qiao Y., Chen X., Chang J. (2018). Effects of upstream and downstream dam operation on the spawning habitat suitability of *Coreius guichenoti* in the middle reach of the Jinsha River. Ecol. Eng..

[45] Ruijing Z. (1998). River Sediment Dynamics.

[46] Ministry of Water Resources of the People’s Republic of China (2021). China Hydrological Yearbook.

[47] China Three Gorges Corporation (2020). Reservoir Application and Power Station Operation Regulation for the Wudongde Hydropower Station on the Jinsha River.

[48] Ruan Y., Tuo Y., Deng Y., Xu X. (2017). Prediction of water temperature in wudongde reservoir and mitigation measures of low-temperature water. Resour. Environ. Yangtze Basin.

[49] Yao W. (2016). Application of the Ecohydraulic Model on Hydraulic and Water Resources Engineering. Ph.D. Thesis.

[50] Yang Z., Tang H., Tao J., Zhao N. (2017). The effect of cascaded huge dams on the downstream movement of *Coreius guichenoti* (Sauvage & Dabry de Thiersant, 1874) in the upper Yangtze River. Environ. Biol. Fishes.

[51] Zhang P., Cai L., Yang Z., Chen X., Qiao Y., Chang J. (2018). Evaluation of fish habitat suitability using a coupled ecohydraulic model: Habitat model selection and prediction. River Res. Appl..

[52] Yu L., Lin J., Chen D., Duan X., Peng Q., Liu S. (2018). Ecological flow assessment to improve the spawning habitat for the four major species of carp of the Yangtze River: A study on habitat suitability based on ultrasonic telemetry. Water.

[53] Zhitang Y., Zhixin L., Bolu Y. (1984). The early development of *Coreius heterodon* and *Coreius guichenoti*. Acta Hydrobiol. Sin..

[54] Kolar C.S., Chapman D., Courtenay W.R., Housel C.M., Williams J.D., Jennings D.P. (2007). Bigheaded Carps: A Biological Synopsis and Environmental Risk Assessment.

[55] Kocovsky P.M., Chapman D.C., McKenna J.E. (2012). Thermal and hydrologic suitability of Lake Erie and its major tributaries for spawning of Asian carps. J. Great Lakes Res..

[56] Stanley J.G., Miley W.W., Sutton D.L. (1978). Reproductive requirements and likelihood for naturalization of escaped grass carp in the United States. Trans. Am. Fish. Soc..

[57] Leslie A.J., Van Dyke J.M., Nall L.E., Miley W.W. (1982). Current velocity for transport of grass carp eggs. Trans. Am. Fish. Soc..

[58] George A.E., Chapman D.C., Deters J.E., Erwin S.O., Hayer C.A. (2015). Effects of sediment burial on grass carp, Ctenopharyngodon idella (Valenciennes, 1844), eggs. J. Appl. Ichthyol..

[59] Garcia T., Zamalloa C.Z., Jackson P.R., Murphy E.A., Garcia M.H. (2015). A Laboratory Investigation of the Suspension, Transport, and Settling of Silver Carp Eggs Using Synthetic Surrogates. PLoS ONE.

